# Dr. Monro on the Milt-Like Tumour of Mucous Membranes
*Morbid Anatomy of the Stomach, &c. 2d Edition.


**Published:** 1830-01-01

**Authors:** 


### XLIX.

### DR. MONRO ON THE MILT-LIKE TUMOUR OF MUCOUS MEMBRANES.*

So many years have elapsed since the first edition of Dr. Monro's work was published, that many points which were noticed then will be feebly remembered, or perhaps entirely forgotten, now. We shall refresh our readers' memory and our own by noticing such portions of this valuable volume as appear to us to be least generally understood. The section on what Dr. Monro has called the milt-like tumour of the mucous membranes appears to us to be in this predicament. Dr. Monro observes that this tumour, though resembling in some respects the "anomalous tumour" of his grandfather, or what is now commonly called fungus hæmatodes, differs from it in several leading particulars. The doctor gives first a description of the disease and then enumerates the points of distinction between it and fungus hæmatodes.

"This species of tumour generally attains so considerable a volume, as to fill, and even distend to an unnatural size, the bowel within which it is contained, as I have seen in the case of the bladder of urine; but, in other cases, this tumour grows from a part only of the mucous membranes.

"The disease does not prove speedily fatal; for I had occasion to attend a patient who laboured under it for two or three years.

"The milt-like tumour, in many respects, resembles the milt of fishes; it is of a pale red colour; and it also is nearly of the same consistence, but rather softer; has an irregular surface, and is covered by a thin membrane, upon which there is a number of vessels filled with blood.

"This species of tumour very readily falls to pieces, and mixes in part with water, forming a turbid mixture; and it becomes somewhat hardened by being put into strong spirits. It ad-

* Morbid Anatomy of the Stomach, &c. 2d Edition.

heres but slightly to the organ from which it grows, by a number of small processes, which insinuate themselves into the thickened villous coat; after the tumour has been detached, the villous coat of the diseased bowel assumes somewhat of a honey-comb appearance, and it is besmeared with several drops of blood, derived from the vessels which supplied the tumour having been torn.

"The bowel from which such a tumour grows, betrays marks of inflammation externally; there is evidently an unnatural determination of blood to the seat of the disease, the blood-vessels upon the peritoneal coat, being not only larger, but also more numerous, than in the healthy state.

"The neighbouring lymphatic glands also participate in the disease, being much larger than in the healthy state; and they are filled with precisely the same milt-like matter. In a case which I had occasion to examine, the bladder was filled with the milt-like matter, and one of the lymphatic glands at the side of it had attained the size of the fist, so that I at first supposed there had been a morbid contraction in the middle of the bladder; but found, upon opening it, that there was no communication between the cavity of the bladder and the swelling connected with it.

"There is another peculiarity in the disease, viz. the emission of a very remarkable and offensive fœtor: the organ containing such a tumour is as much discoloured, and emits as fœtid a smell, as the same bowel which had been exposed to the air for several days.

"The veins distributed on the mucous membrane, in the vicinity of the tumour, are considerably enlarged and distended with blood."

The differences noted by our author between this and the "anomalous tumour" are as follows:

"1st. The anomalous tumour of my Grandfather differs, in its situation, from the milt-like tumour; the former has been found in all the tissues of the body, the latter in the mucous membranes only.

"The anomalous tumour is sometimes connected with the periosteum, the capsular ligaments, especially with that of the hip-joint, the peritoneum, the liver, spleen, ovarium, and uterus, and with the albuginea and vaginal coats of the testicle; with the coats of the optic nerve, and sclerotic coat of the eye.

"In short, the anomalous tumour is not confined to any one texture, but is common to all; it begins as a distinct elastic tumour, without fluctuation, and some parts of it feel harder than others. In its progress it bursts, and a soft dark purple-coloured fungous excrescence, which bleeds profusely, rises from the centre, and soon increases very rapidly in size.

"The milt-like tumour is much softer than the anomalous; before it bursts, it has not the same purple colour and firm elastic feeling as the anomalous tumour, which Dr. BURNS has compared to a sponge tied up very tightly in a piece of bladder; and I have never observed any appearance of fungous ulceration of the milt-like tumour, nor the same inequality in the consistence of different parts of it, as in the fungus hæmatodes.

"The tumour is nearly of an uniform consistence in every part, and its lobes are not so distinct as those of the anomalous, which are separated from each other by membranes.

"The milt-like tumour is, in colour and consistence, uniform in every part; but the section of the anomalous tumour exhibits, in its parts, a different colour, and also a different consistence, some portions being as soft as brain, others as hard as the yolk of a boiled egg, and others like cartilage; besides, there are cavities, of different sizes and forms, within the tumour, full of a fluid tinged with blood.

"The disease I have been endeavouring to describe, appears only in advanced life; but the anomalous tumour is, in many instances, a disease of infancy, of childhood, and of the meridian of life."

Dr. Monro details the particulars of an interesting case communicated to him, along with a preparation of the morbid parts, by Dr. Anderson of Leith. The disease was situated at the great end of the stomach, and was accompanied during life by the ordinary symptoms of malignant disease of that organ; namely, sickness after eating, pyrosis, emaciation, disordered state of the intestinal canal. Dr. Anderson observes in his account of the case—"To me it appears an example of the medullary sarcoma, so well described by Mr. Abernethy."

Our author remarks that he had occasion to visit along with Mr. Allan, surgeon, a man who was afflicted with this disease in his bladder, and who for ten months before his death suffered the most excruciating agony, and was often convulsed. Dr. Monro's father also met with a similar tumour in the bladder many years ago. The following case is adduced as a good example of the milt-like tumour of the stomach.

*Case.* John Leishman, æt. 35, admitted into the Royal Infirmary of Edinburgh on the 22d of December, 1824, much emaciated and hectic, with a large tumour of a livid colour in the umbilical region. In the center of the tumour was an ulceration, which discharged a foul greenish matter, and below was a second smaller ulcerated point; there was much inflammation and extreme sensibility of the surrounding integuments. He stated that for several years he had been addicted to habits of intemperance, and after a debauch he first experienced pains in the stomach of a few days' duration. In April, 1824, these became more permanent, but no distinct account of their nature could be obtained. Fourteen weeks before admission he was suddenly attacked with intense pain in the umbilical region, which in four days was succeeded by inflammation and swelling. Four weeks before admission the ulceration commenced, and since that time the pain had been relieved. He seems to have died in a very short time, for the next mention made of the patient is that of his

"*Dissection.* The integuments were removed, and, as it was discovered that the tumour had extensive adhesions, the whole mass was dissected from the abdomen, and laid upon the table. After removing the small intestines, the ascending and descending colon, the spleen and the kidneys, all of which were natural, the diseased structure, with its connections, became apparent. The tumour involved the stomach, liver, pancreas, duodenum, and the transverse arch of the colon. The stomach was laid open, and its villous coat presented a high degree of vascularity and thickening. Towards the pyloric extremity a number of vascular fungi were observed, and, on passing the hand under these, in the direction of the external orifice of the tumour, the finger passed through a sinuous cavity, and presented at the external opening. The tumour itself exhibited, towards its circumference, a cartilaginous structure, but its centre was sloughy, and yielded a highly offensive fetor. The liver was enlarged, and contained a number of circumscribed white tubercles, of the magnitude of nuts and walnuts, the texture of which was soft, and yielded a milky fluid on pressure. Similar tubercles, of less size, pervaded the mesentery and omentum. The cavity of the abdomen contained about two or three pounds of bloody serum."

In another case of milt-like tumour of the stomach, there were the following symptoms:—The patient's aspect was pale and he was emaciated; the abdomen was tender in the epigastric region; sometimes he vomited after every meal, but at other times there was no vomiting for two or three days; occasional diarrhœa, with tormina; pulse and tongue natural. The degree of pain attending fungus hæmatodes is, according to our author, greater than that accompanying the milt-like tumour.

So much for this remarkable disease of which we have never witnessed an example. We must confess, however, that the distinctions between it and fungus hæmatodes do not seem to us to be so broad and characteristic as Dr. Monro imagines. The appearances of the latter disease are not the same in every instance, no more are the symptoms. In one case there will be much pain, in another there will be little or none—frequently the patient is young, but he is also not unfrequently advanced in life—sometimes the progress of the disease is rapid, sometimes it is very slow. Again, with regard to the local characters of fungus hæmatodes, we can say with confidence that we never saw two samples of the tumour that did not differ in some respects from each other. In one there is the hæmatoid appearance, in another there is not—in some instances the tumour is very soft, in others so firm as to approach, in a great degree, to the consistency of scirrhus—occasionally the colour is dark, more commonly of brain-like whiteness, and so on. In the account of the case reported as a good example of the milt-like tumour of the stomach, the description of the circumscribed white tubercles in the liver answers exactly to that of the medullary disease of that organ. We mention these circumstances, not to throw discredit on the tact and discrimination of Dr. Monro, both of which he possesses in an eminent degree, but to inspire caution in the classification of these malignant diseases. We have before observed, and we need not go over the ground again, that even the two great varieties, scirrhus and fungus hæmatodes, evince a very marked disposition to run into and blend with one another.

